# Sugar supplementation enhances biofilm formation and extracellular polysaccharides production in *Sulfobacillus acidophilus*

**DOI:** 10.3389/fmicb.2026.1838761

**Published:** 2026-05-19

**Authors:** Javiera Pizarro, Luna López, Mauricio Díaz, Moritz Gansbiller, Sergio A. Álvarez, Jochen Schmid, Mario Vera

**Affiliations:** 1 Instituto de Ingeniería Biológica y Médica, Facultades de Ingeniería, Medicina y Ciencias Biológicas, Pontificia Universidad Católica de Chile, Santiago, Chile; 2 Institute of Molecular Microbiology and Biotechnology, University of Münster, Münster, Germany; 3 Laboratorio de Microbiología, Departamento de Bioquímica y Biología Molecular, Facultad de Ciencias Químicas y Farmacéuticas, Universidad de Chile, Santiago, Chile; 4 Departamento de Ingeniería de Minería, Pontificia Universidad Católica de Chile, Santiago, Chile

**Keywords:** biofilm, epifluorescence microscopy, extracellular polysaccharide, lectin binding, sugar transporters, *Sulfobacillus*

## Abstract

*Sulfobacillus* is an acidophilic bacterial genus that plays a key role in bioleaching of metal sulfides at moderately thermophilic temperatures (45–55 °C), owing to its mixotrophic metabolism and capacity to form biofilms. Biofilm development depends on an extracellular polymeric substances (EPS) matrix, which is largely composed of polysaccharides. However, the composition and function of polysaccharides in sulfobacilli remain poorly characterized. A genome-wide survey identified a diverse repertoire of putative sugar uptake transporters, belonging to three distinct families. We demonstrate that supplementation with glucose, fructose, xylose or sucrose increases biofilm formation and extracellular polysaccharides production in *Sulfobacillus acidophilus* DSM 10332ᵀ. Enhanced biofilm formation was quantified using large-scale, in-depth image analysis. Fluorescent lectin binding analysis revealed a concomitant increase in extracellular polysaccharides abundance, while HPLC-UV-ESI-MS confirmed a quantitative rise in secreted polysaccharides under sugar-supplemented conditions. Detailed compositional analysis showed that the extracellular polysaccharides produced by *S. acidophilus*^T^ are predominantly composed of glucose and mannose residues. Overall, our findings indicate that sugar supplementation stimulates biofilm development in *S. acidophilus*, likely by enhancing extracellular polysaccharides biosynthesis. This study provides the first detailed compositional characterization of *S. acidophilus*^T^ extracellular polysaccharides and establishes *Sulfobacillus* as a promising model for investigating sugar uptake and carbon cycling in bioleaching systems, as members of this genus can also grow without the supplementation of sugars.

## 1 Introduction

Biomining is the process of recovering metal cations from mineral ores through the metabolic activity of extremely acidophilic microorganisms that oxidize ferrous iron and/or reduced inorganic sulfur compounds (RISCs) (Vera et al., 2022). In contrast to the well-studied mesophilic, chemolithotroph genera such as *Acidithiobacillus* and *Leptospirillum*, *Sulfobacillus* comprises moderately thermophilic, mixotrophic, non-motile bacteria (Norris et al., 1996). Members of this genus are capable of oxidizing ferrous iron, elemental sulfur and RISCs, and can also reduce ferric iron (Christel et al., 2018). *Sulfobacillus* species can grow autotrophically via the Calvin–Benson–Bassham cycle (Caldwell et al., 2007). They also exhibit mixotrophic growth utilizing simple sugars, glycogen and other organic compounds (Panyushkina and Muravyov, 2023) to sustain cellular growth. To date, six species of the genus *Sulfobacillus* have been described: *Sulfobacillus thermosulfidooxidans*, *Sulfobacillus acidophilus*, *Sulfobacillus sibiricus*, *Sulfobacillus thermotolerans*, *Sulfobacillus benefaciens* (Justice et al., 2014) and *Sulfobacillus harzensis* (Zhang et al., 2021).

*Sulfobacillus*, like other bioleaching bacteria, can aggregate with one another and attach to mineral surfaces, forming monolayer biofilms (Bellenberg et al., 2018; Christel et al., 2018). Biofilm formation is essential for interactions between bioleaching microorganisms and mineral substrates and strongly influences bioleaching rates of metal sulfides such as pyrite and chalcopyrite (Vera et al., 2022). During biofilm development, cells secrete biopolymers that form a matrix of extracellular polymeric substances (EPS), which embeds and stabilizes cellular aggregates. The composition of EPS varies among species and environmental conditions but typically includes polysaccharides (PS), proteins, lipids, and extracellular DNA (Flemming et al., 2016). PS found in the EPS are synthesized intracellularly and are subsequently exported to the extracellular space. In *Acidithiobacillus ferrooxidans* ATCC 23270^T^, supplementation with glucose or mannose resulted in increased capsular PS production during biofilm formation on pyrite, suggesting that acidophiles may scavenge environmental sugars to support PS biosynthesis during biofilm development (Bellenberg et al., 2012).

The biofilm matrix can be examined using epifluorescence microscopy (EFM). Microbial cells are commonly visualized using protein, lipid or nucleic acid binding fluorescent dyes, while PS within the matrix can be detected by means of a Fluorescent Lectin Binding Analysis (FLBA), in which commercially available lectins bind glycoconjugate residues, with varying degrees of specificity (Neu and Kuhlicke, 2017). Lectins have been widely used to visualize biofilms formed by acidophilic microorganisms, revealing distinct and specific binding patterns. FLBA has been applied to biofilms of acidophilic microorganisms, where lectins such as AAL (*Aurelia Arantia* Lectin) and ConA (Concanavalin A, *Canavalia ensiformis* hemagglutinin) bound to the PS of *Ferroplasma acidiphilum* DSM 28986, *Sulfolobus metallicus* DSM 6482ᵀ, and *Acidianus* sp. DSM 29099, indicating the presence of fucose, mannose and glucose residues. Certain lectins primarily bound to capsular PS, whereas others produce more diffuse, colloidal binding signals (Zhang et al., 2015). In bioleaching bacteria, ConA has been shown to bind to *A. ferrooxidans*ᵀ, confirming the presence of glucose and mannose-containing residues within the extracellular PS (Bellenberg et al., 2012). In addition, lectins AIA (*Artocarpus integrifolia*) and GS-I (*Griffonia simplififolia* lectin I) have shown strong binding to *Leptospirillum ferriphilum*^t^, suggesting the presence of galactose and N-acetyl galactosamine-containing residues (Neu and Kuhlicke, 2017). In addition to structural characterization, EFM can be used to quantify microbial colonization and biofilm formation. Bioleaching bacteria colonize mineral surfaces heterogeneously; consequently, this intrinsic spatial variability requires a large-scale image analysis to obtain statistically robust measurements of colonization and biofilm development (Bellenberg et al., 2018; Rossoni et al., 2024).

Several transport systems mediate sugar uptake in bacteria, including (i) the phosphoenolpyruvate-dependent sugar phosphotransferase system (PTS), (ii) the ATP-binding cassette (ABC) transporters of the carbon uptake transporter subfamilies CUT-1 and CUT-2, and (iii) members of the Major Facilitator Superfamily (MFS) (Jeckelmann and Erni, 2020). A functional PTS system requires the cytoplasmic proteins enzyme I (EI) and histidine-containing phosphocarrier protein (Hpr), together with a variable set of proteins forming a sugar-specific enzyme II (EII) complex (Saier, 2015). In contrast, ABC transporters, called “Carbon Uptake Transporters” CUT-1 and CUT-2 subfamilies, comprise three core components: a substrate-binding protein (SBP), a nucleotide-binding protein (NBP) and a transmembrane (TM) domain. In CUT-1 systems, the TM domain is encoded by two genes, whereas CUT-2 systems contain a single TM-encoding gene (Schneider, 2001). MFS transporters are encoded by a single gene and exhibit a highly conserved architecture consisting of 12 transmembrane helices, with a typical length of approximately 400–500 amino acids (Drew et al., 2021). In addition to sugar uptake, bacteria can import nucleobases for nucleic acid biosynthesis. Transporters responsible for nucleobase uptake belong to the Nucleobase: Cation Symporter (NCS) family, which is subdivided into the NCS-1 and NCS-2 subfamilies. Similar to members of the MFS family, NCS transporters are encoded by a single gene and exhibit the conserved architecture of 12 transmembrane (TM) helices with the typical length of approximately 400–600 amino acids (Weyand et al., 2008). In addition to a potential capacity for importing entire nucleobases, these may represent extra pools for sugars. At highly acidic environments DNA is depurinated, and acid conditions can enhance hydrolysis of glycosidic bonds between the nitrogenous base and the pentose sugars.

Despite their ecological relevance, relatively little is known regarding sugar transport and utilization in *Sulfobacillus* species. *Sulfobacillus acidophilus* strain NAL: DSM 10332^T^ is able to grow autotrophically on ferrous iron, elemental sulfur or pyrite, but is also able to grow heterotrophically using yeast extract as the sole substrate (Norris et al., 1996). Mixotrophic growth of this strain has been shown to occur in ferrous iron cultures supplemented with glucose. Other studies have demonstrated that supplementation with glucose and α-glucans supports heterotrophic growth of other species of *Sulfobacillus* (Panyushkina and Muravyov, 2023). All these studies focused primarily on planktonic growth in the presence of organic substrates and did not address whether these sugars are assimilated into PS biosynthesis. Justice et al. (2014) analyzed the complete genomes of five environmental *Sulfobacillus* spp. and identified genes encoding the glycolytic pathway, pyruvate oxidation, and a complete non-oxidative branch of the pentose phosphate pathway, enabling interconversion of pentoses and hexoses. The genomic survey also revealed the presence of genes encoding putative sugar ABC transport proteins. Nevertheless, these transport systems were not further characterized, nor was their functionality experimentally validated. We therefore investigated whether exogenous sugars can be imported and channeled into extracellular PS biosynthesis, thereby promoting not only planktonic growth but also biofilm development.

In this study, we investigated biofilm formation in *S. acidophilus*^T^ using a combination of bioinformatics and experimental approaches. First, we conducted an in-depth *in silico* analysis to identify putative sugar transporters encoded in the genome. We then examined the effects of sugar supplementation on biofilm formation using EFM microscopy and FLBA. Finally, we assessed whether imported sugars contribute to extracellular PS biosynthesis and performed chemical characterization of the monomeric composition of the produced PS using HPLC-UV-ESI-MS.

## 2 Materials and methods

### 2.1 Bioinformatic analysis

Complete genome sequences of *S. thermosulfidooxidans* DSM 9293^T^ and *S. acidophilus* DSM 10332^T^ were searched against the Transporter Classification Database (TCDB) (Saier et al., 2021),^1^ using GBlast. Representative transporters from TCDB families of interest were used as query sequences. All hits with a bit score greater than 100, relative to the query sequences, were retained and subsequently subjected to manual curation to identify putative sugar transporters.

The membrane topology of all retrieved sequences was predicted using the DeepTMHMM online tool (Hallgren et al., 2022).^2^ Signal peptide presence and cleavage sites were identified using SignalP 6.0 (Teufel et al., 2022).^3^ For MFS and NCS transporters, sequences were retained only if they exhibited the expected topology of 12 TM α-helices (Quistgaard et al., 2016). Transmembrane subunits of the ABC transporters were selected based on the presence of the expected topology of three or six TM α-helices (Schneider, 2001).

To infer the putative function of the retrieved transporters, multiple databases and search tools were employed. Conserved domain searches were performed using the CD-Search interface of the NCBI Conserved Domain Database (CDD) (Marchler-Bauer and Bryant, 2004)^4^ and in the Pfam database (Mistry et al., 2021,^5^ both accessed in November 2023), applying an e-value threshold of 0.01 with composition-based score adjustment. Profile and motif searches were conducted using ScanProsite (De Castro et al., 2006),^6^ and specific motif occurrences were identified using FIMO from the MEME Suite (Grant et al., 2011).^7^

Genomic context and functional annotations were analyzed using the RAST (Rapid Annotation using Subsystem Technology) server (Aziz et al., 2008),^8^ which provided subsystem-based annotations for the complete genomes *S. acidophilus*^t^ and *S. thermosulfidooxidans*^t^.

### 2.2 Biofilm formation assays

Biofilm formation was assessed in 6-well plates. Each well was inoculated with *S. acidophilus* DSM 10332^T^ at a cell density of 10^7^ cells/mL. Cells were cultured in K medium with the following composition: 1 g/L of (NH_4_)_2_SO_4_, 0.1275 g/L of NaH_2_PO4·H_2_O, 0.10 g/L of MgSO_4_·7H_2_O, and 0.021 g/L of CaCl_2_·2H_2_O (Merino et al., 2016). The medium pH was adjusted to 1.7 using 5 M H_2_SO_4_. Fe (II) was added at 2 g/L, yeast extract at 0.02% w/v, and elemental sulfur (S^0^) pearls at 1% w/v. Sugars (Sigma-Aldrich), were supplemented to a final concentration of 1 mM. In separate experiments, DNA (Sigma-Aldrich) was added at 1 ng/μL. Plates were incubated under static conditions at 37 °C in a Memmert® IN75 incubator for 96 h to allow biofilm development. Following incubation, several sulfur pearls were collected from the well bottom for EFM microscopy (See below).

Planktonic growth was monitored daily. Cell counts were determined using an improved Neubauer® chamber (0.02 mm depth, Marienfeld GmbH & Co, Germany). Medium pH and oxidation–reduction potential (ORP) were measured using a dual-channel laboratory pH/mV meter (HI 5222, Hanna Instruments).

### 2.3 Sample preparation for epifluorescence microscopy

Elemental sulfur pearl samples were collected and immediately stored at −20 °C. Prior to staining, samples were thawed and washed three times with Tris-EDTA (TE) buffer (10 mM Tris HCl, pH 8.0, 1 mM EDTA), then stained with 1.2 μM 4′,6-diamidino-2-phenylindole (DAPI; Sigma-Aldrich) for 30 min.

For FLBA, samples were washed with filtered distilled water and fixed in 4% w/v paraformaldehyde (PFA) for 30 min. Fixed samples were washed again with distilled water and stored at 4 °C. Prior to visualization, samples were incubated with DAPI as described above, followed by incubation with FITC- or TRITC-conjugated lectins for 30 min. Lectins were applied at a final concentration of 100 ng/mL, according to the manufacturer’s instructions. Fluorescently labeled lectins were obtained from Vector Labs or Invitrogen.

### 2.4 Epifluorescence microscopy

Biofilms of *S. acidophilus*^T^ formed on sulfur pearls were visualized using the Zeiss® Axio Observer Z1/7 inverted EFM microscope equipped with a 40× objective (Zeiss Objective EC Plan-Neofluar 40×/0.75 M27) and a motorized scanning stage (130 × 85). Images were acquired using the z-stack function, with 15–55 optical slices, depending on pearls morphology, and a slice spacing of 0.55 μm. A 4 × 4 tiles mode was applied, resulting in 16 fields per image. Each 16 tiles image measured 5,135 × 3,841 pixels, corresponding to a physical area of 947.5 μm × 708.8 μm. DAPI-stained samples were imaged using Zeiss filter set 49 (emission peak 465 nm) and bright-field reflected light imaging was used for sample localization.

For visualization of extracellular glycoconjugates by FLBA, both individual and tiled images were acquired with the same 40× objective. Additionally, a Zeiss Filter set with an emission peak at 519 nm and/or 581 nm were used.

### 2.5 Microscopy image processing

Z-stack tile images were compiled using the *Extended Depth of Focus* function in Zen® 3.0 (Blue Edition). Processing was performed in contrast mode without alignment, with flash decay correction enabled, a wavelength scale of 7, smoothing set to 11, and a reconstruction parameter of 0.15. For images acquired for large-scale quantitative analysis, fluorescence channel histograms were automatically adjusted using the Best Fit function, whereas images used for FLBA were manually adjusted. Regions of Interest (ROIs) were generated using the ‘Create Image Split and Subset’ function, and size bars were added within the same software. All subsequent quantitative analyses were carried out using images from the DAPI fluorescence channel.

Image analysis was conducted in JupyterLab using a modified version of the image-processing notebook described by Bellenberg et al. (2018), implemented in Python 3.0 within an Anaconda (conda-forge) environment. Each image was subjected to contrast enhancement and sharpening filters, followed by background extraction using a rolling-ball (“ballrolling”) algorithm. From the binary mask, the outline of each microcolony was delineated.

### 2.6 Quantitative analysis and statistics

Each experimental condition included in the large-scale image analysis was done in biological triplicate. For each replicate, four sulfur pearls were examined, and for each pearl, four independent 4 × 4 tile images were acquired. This resulted in 64 tiles per pearl and 256 tiles per biological replicate, yielding a total of 768 tiles analyzed per experimental condition. For the control condition, data were obtained from two biological replicates, each comprising three technical replicates, resulting in a total of 1,536 tiles analyzed.

EFM images were processed using a custom Python-based analysis pipeline, enabling the extraction of quantitative parameters relevant to biofilm formation. Specifically, colony density (colonies/mm^2^) and the percentage of colonized surface area were calculated. Quantitative results were visualized using GraphPad Prism with datasets grouped according to experimental condition. Because the data did not conform to a Gaussian distribution, statistical comparisons were made using the non-parametric Kruskal-Wallis test to assess overall differences among conditions. When significant differences were detected, Dunn’s post-hoc test was applied to identify pairwise differences between groups. To ensure that the image analysis was statistically representative, coefficients of variation (CV) were calculated for each biological replicate, as described by Bellenberg et al. (2018). For each condition, four randomly selected subsets of images were generated with increasing group sizes (1, 2, 9, 18, 36, and 72 images) until CV values stabilized below 10%. Low CV values indicate that enough images had been analyzed to reliably assess biofilm formation.

### 2.7 Extracellular polysaccharide production and recovery

To promote PS production, *S. acidophilus*^T^ was cultivated in shaken flasks at 150 rpm and 50 °C for 168 h. The inoculum was added at a density of 10^7^ cells/mL. Cells were cultivated in Kmedium as previously described, supplemented with 0.02% (w/v) yeast extract and 0.5% (w/v) sulfur powder. Monosaccharides (glucose, fructose and xylose) were added to a final concentration of 5 mM, whereas the disaccharide sucrose was added at 2.5 mM. Sugar concentrations were increased relative to the biofilm formation assays to compensate for the higher cell densities reached in shaken cultures. Planktonic cell densities were determined daily, as described previously. All cultures were prepared in biological triplicates, and statistically significant differences in growth curves were assessed using two-way ANOVA in GraphPad Prism. Residual sugar concentrations in the culture medium were monitored daily using the Dubois colorimetric method (Dubois et al., 1951).

Cell-free supernatants were obtained by centrifugation at 9,000×*g* for 10 min and subsequent filtration through 0.22 μm syringe filters. PS were precipitated by adding two volumes of analytical-grade isopropanol to one volume of supernatant, followed by mixing and incubation at −20 °C overnight. Precipitated PS were collected by centrifugation under the same conditions (9,000×*g*, 10 min).

### 2.8 Extracellular PS analysis by HPLC-ESI-MS/MS

For the analysis of PS monomer composition, precipitated samples were dissolved in 1 mL of water at pH 2.4. Alternatively, cell-free supernatant was directly used for analysis. The monomer analysis was done according to Rühmann et al (2014). For the hydrolysis, 20 μL of the sample were mixed with 20 μL of 4 M trifluoroacetic acid (TFA) in a 96-well PCR plate, which was sealed with a TPE cap mat and clamped between two custom-made metal plates to prevent evaporation. Hydrolysis was carried out in a sand bath at 121 °C for 90 min. After hydrolysis, samples were neutralized to approximately pH 8.0 using 3.2% NH_4_OH. To check successful neutralization, unused neutralized sample volumes were tested with a phenol red solution (× mg phenol red in 50 mL y % ethanol). 25 μL of neutralized hydrolysate was mixed with 75 μL of PMP-derivatization mixture (125 mg 1-phenyl-3-methyl-5-pyrazolone, 7 mL MS-grade methanol, 437.5 μL 3.2% NH_4_OH and 3.0625 mL ultrapure Water) in a 96-well PCR plate and incubated for 99 min at 70 °C in a PCR thermal cycler. 20 μL of derivatized sample was mixed with 130 μL of 19.23 mM acetic acid and submitted to LC–MS analysis. As standards, a combination of different monosaccharides at concentrations from 2 to 50 mg L^−1^ were derivatized in the same manner of sample preparation.

HPLC-ESI-MS/MS analyses were carried out on a Waters Acquity UPLC, equipped with a UV Detector at 245 nm and a Bruker Amazon Speed ESI-IT-MS. HPLC separation was carried out with a Nucleodur C18 Gravity column (100 × 2 mm) at 50 °C and a flow rate of 0.6 mL min^−1^ The gradient of Solvents A (Acetonitrile) and Solvent B (5 mM ammonium acetate buffer pH 5.6 with 15% Acetonitrile) was as follows: 0–6 min 99% B – 95% B; 6–8 min 95–82% B; 9–9.3 min 82–60% B; 9.3–11.3 min 60% B; 11.3–11.5 min 60–99% B; 11.5–13 min 99% B. ESI parameters were set to positive mode with a capillary voltage of 4 kV, end plate offset 500 V, dry gas temperature 325 °C, dry gas flow 8 L min^−1^, nebulizer pressure 40 kPa. Between the UV-Detector and the ESI, a flow 20:1 flow split was used. Ion-trap parameters were set to ultra scan mode (32,000 m/z/s) from 50 to 1,000 m/z, ICC target 200,000, max. Accumulation time 50 ms, 4 averages, auto-MS^n^ with a number of precursor ions of 2. Data was evaluated with QuantAnalysis version 5.3 using the respective extracted ion chromatograms (EIC).

## 3 Results

### 3.1 *Sulfobacillus* possesses several sugar transporters belonging to three different families

**Figure 1:**
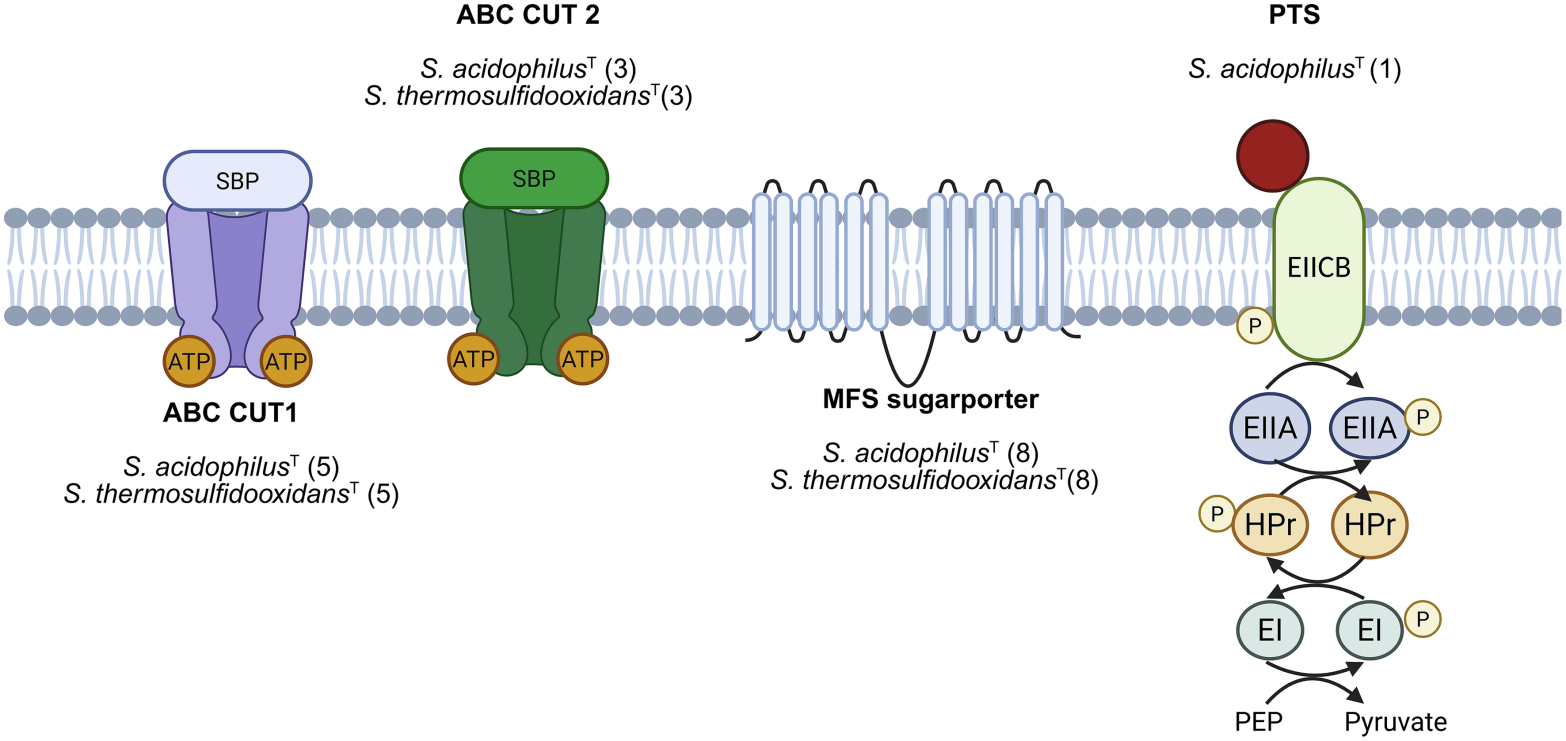
Putative sugar transporters found in the genomes of *S. acidophilus*^T^ and *S. thermosulfidooxidans*^T^. Five putative ABC CUT1-type transporters and three putative ABC CUT2-type transporters were identified in the genomes of both *S. acidophilus*^T^ and *S. thermosulfidooxidans*^T^. In addition, eight putative sugar MFS transporters were detected in each species. A single putative PTS system was identified exclusively in the genome of *S. acidophilus*^T^. With respect to nucleobases transport, seven putative NCS-1 transport systems were identified in *S. acidophilus*^T^, whereas 10 such systems were detected in *S. thermosulfidooxidans*^T^. Created in BioRender. Pizarro, J. (2026) https://BioRender.com/mpasqza.

A comparative bioinformatic analysis of the type strains of *S. acidophilus*^T^ and *S. thermosulfidooxidans*^T^ was performed to identify putative sugar uptake transport systems. In total, 17 and 16 putative sugar uptake transporters were identified for *S. acidophilus*^T^ and *S. thermosulfidooxidans*^T^, respectively. Since nucleobases also contain sugars, we included the search for NCS transporters. We identified 10 and 7 putative NCS-1 transport systems for nucleobases import in the genomes of *S. acidophilus*^T^ and *S. thermosulfidooxidans*^T^, respectively. Notably, the genome of *S. acidophilus*^T^ encodes a complete PTS system, predicted to mediate fructose import, which is absent in *S. thermosulfidooxidans*^T^ (Figure 1). After this search, we decided to study sugar addition to *S. acidophilus*^T^ and to follow biofilm formation and extracellular polysaccharide production.

#### 3.1.1 MFS transporters

In both *S. acidophilus*^T^ and *S. thermosulfidooxidans*^T^ eight coding sequences were identified as putative MFS sugar transporters (Table 1). Given the generally broad substrate specificity of MFS, it was not possible to assign a single, definitive substrate to each transporter. To identify potential orthologous relationships between the putative MFS transporters of *S. thermosulfidooxidans*^T^ and *S. acidophilus*^T^, a Bidirectional Best Hit (BBH) analysis was performed. Based on BBH criteria, four of the putative MFS transporters present in both genomes were classified as orthologous (Table 1).

**Table 1:** Sequences for the putative transporters found in the genomes of *S. acidophilus*^T^ y *S. thermosulfidooxidans*^T^.

MFS sugarporter *S. acidophilus^T^*	MFS sugarporter *S. thermosulfidooxidans^T^*
AEW03687.1, AEW03843.1, AEW04311.1, *AEW04366.1* (O1), AEW04492.1, *AEW06192.1* (O2), *AEW06836.1* (O3), *AEW06866.1* (O4)	*SMC02092.1* (O2), SMC03406.1, *SMC03458.1* (O4), *SMC03679.1* (O1), SMC04707.1, SMC06847.1, SMC07961.1, *SMC08103.1* (O3)
ABC CUT-1 *S. acidophilus^T^*	ABC CUT-1 *S. thermosulfidooxidans^T^*
Glucose/Mannose	Glucose/Mannose
*AEW06695.1* (O5), *AEW06696.1* (O6), *AEW06697.1* (O7)	*SMC02421.1* (O7), *SMC02424.1* (O6), *SMC02425.1* (O5)
Xylose transporter	Glycerol transporter
AEW05613.1, AEW05614.1, AEW05615.1	SMC02096.1, SMC02097.1, SMC02098.1
Trehalose transporter	Galactooligosacharides transporter
AEW05654.1, AEW05655.1, AEW05656.1	SMC03611.1, SMC03613.1, SMC03615.1
No substrate assigned	No substrate assigned
AEW05692.1, AEW05693.1, AEW05694.1	SMC04071.1, SMC04073.1, SMC04077.1
No substrate assigned	No substrate assigned
AEW05739.1, AEW05740.1, AEW05741.1	SMC08218.1, SMC08219.1, SMC08220.1
ABC CUT-2 *S. acidophilus^T^*	ABC CUT-2 *S. thermosulfidooxidans^T^*
Ribose transporter	Ribose transporter
*AEW05601.1* (O8), *AEW05602.1* (O9), *AEW05603.1* (O10)	*SMC03773.1* (O8), *SMC03777.1* (O9), *SMC03779.1* (O10)
Nucleoside transporter	Nucleoside transporter
*AEW06309.1* (O11), *AEW06310.*1 (O12), AEW06311.1 (O13), *AEW06312.1* (O14)	*SMC05763.1* (O14), *SMC05769.1* (O13), *SMC05771.1* (O12), *SMC05772.1* (O11)
No substrate assigned	No substrate assigned
AEW05664.1, AEW05665.1, AEW05666.1	SMC04677.1, SMC04682.1, SMC04684.1
PTS fructose transporter *S. acidophilus^T^*	PTS *S. thermosulfidooxidans^T^*
AEW06621.1, AEW06622.1, AEW06623.1, AEW06624.1, AEW06625.1, AEW0662161	–
NCS-1 *S. acidophilus^T^*	NCS-1 *S. thermosulfidooxidans^T^*
AEW03658.1, AEW03975.1, AEW04998.1, *AEW05769.1* (O15), *AEW05778.1* (O16), *AEW05805.1* (O17), *AEW06333.1* (O18)	*SMC03286.*1 (O17), *SMC03685.1* (O15), SMC04878.1, SMC05746.1, *SMC05151.1* (O18), SMC05779.1, SMC05783.1, *SMC06926.1* (O16), SMC06946.1, SMC07038.

All putative sequences involved in sugar uptake systems identified in both genomes are shown in this table. Putative substrates were assigned to specific ABC CUT-1 and CUT-2 subtypes based on conserved domain annotations. Transporter pairs classified as orthologs are annotated with the letter “O” and a number ranging from 1 to 18 in order of appeacrence in *S. acidophilus*^T^. Transporters classifies as orthologs are shown in italics.

#### 3.1.2 ABC transporters

Members of the subfamilies CUT-1 and CUT-2 were identified in the genomes of *S. acidophilus*^T^ and *S. thermosulfidooxidans*^T^. Five complete CUT-1 systems were detected in each species. In both genomes, genes encoding the NBP subunit were not located in close genomic proximity to the corresponding CUT-1 transporter operons. This genomic organization is characteristic for the CUT-1 subfamily, as NBPs are frequently encoded elsewhere in the genome and shared among multiple transport systems (Tsujibo et al., 2004). The putative NBP sequences identified in each genome were orthologous according to BBH analysis and exhibited a highly conserved primary sequence spanning the phosphate-binding loop (Walker A) and the magnesium-binding site (Walker B) (Schneider and Hunke, 1998), which are canonical motifs required for nucleotide binding activity. Notably, putative CUT-1 transporters predicted to mediate trehalose and xylose uptake were identified exclusively in the genome of *S. acidophilus*^T^, whereas putative CUT-1 type transporters for glycerol and galactooligosaccharide were present only in *S. thermosulfidooxidans*^T^. These differences suggest species-specific sugar uptake capabilities within the genus *Sulfobacillus*.

Three complete CUT-2 transport systems with genes encoding the NBP, substrate-binding protein (SBP), and TM subunits organized adjacently within the genome. In both species, one CUT-2 system was classified as a ribose transporter based on specific CD-Search matches to ribose-binding substrate proteins. BBH analysis indicated that the ribose transporters from both species are orthologs. In addition, putative CUT2-type nucleoside transporters were detected in both genomes and were likewise identified as orthologs (Table 1).

#### 3.1.3 PTS transporters

In *S. thermosulfidooxidans*^T^, three sequences homologous to the three-domain Dha KLM PTS system of *E. coli* were identified. In this system, dihydroxyacetone (DHA), a three-carbon sugar, is phosphorylated during uptake. DhaK functions as the catalytic subunit responsible for DHA phosphorylation, DhaL acts as a regulatory subunit, and DhaM serves as the phosphoryl donor (Gutknecht et al., 2001). Despite their homology, these sequences were not considered to constitute a complete DhaKLM PTS system, as the sequence homologous to DhaM was substantially shorter than expected. In contrast, the genome of *S. acidophilus*^T^ encodes a complete putative fructose-specific PTS system, featuring fused EIIB and EIIC domains (Table 1).

### 3.2 Supplementation with sugars enhances *S. acidophilus*^T^ biofilm formation on S^0^

Biofilm formation on elemental sulfur and sulfide minerals by bioleaching bacteria is highly heterogeneous, making quantitative analysis challenging and image-intensive. To address this, we employed a semi-automated high-throughput image analysis workflow to quantitatively assess biofilm formation under different experimental conditions.

**Figure 2:**
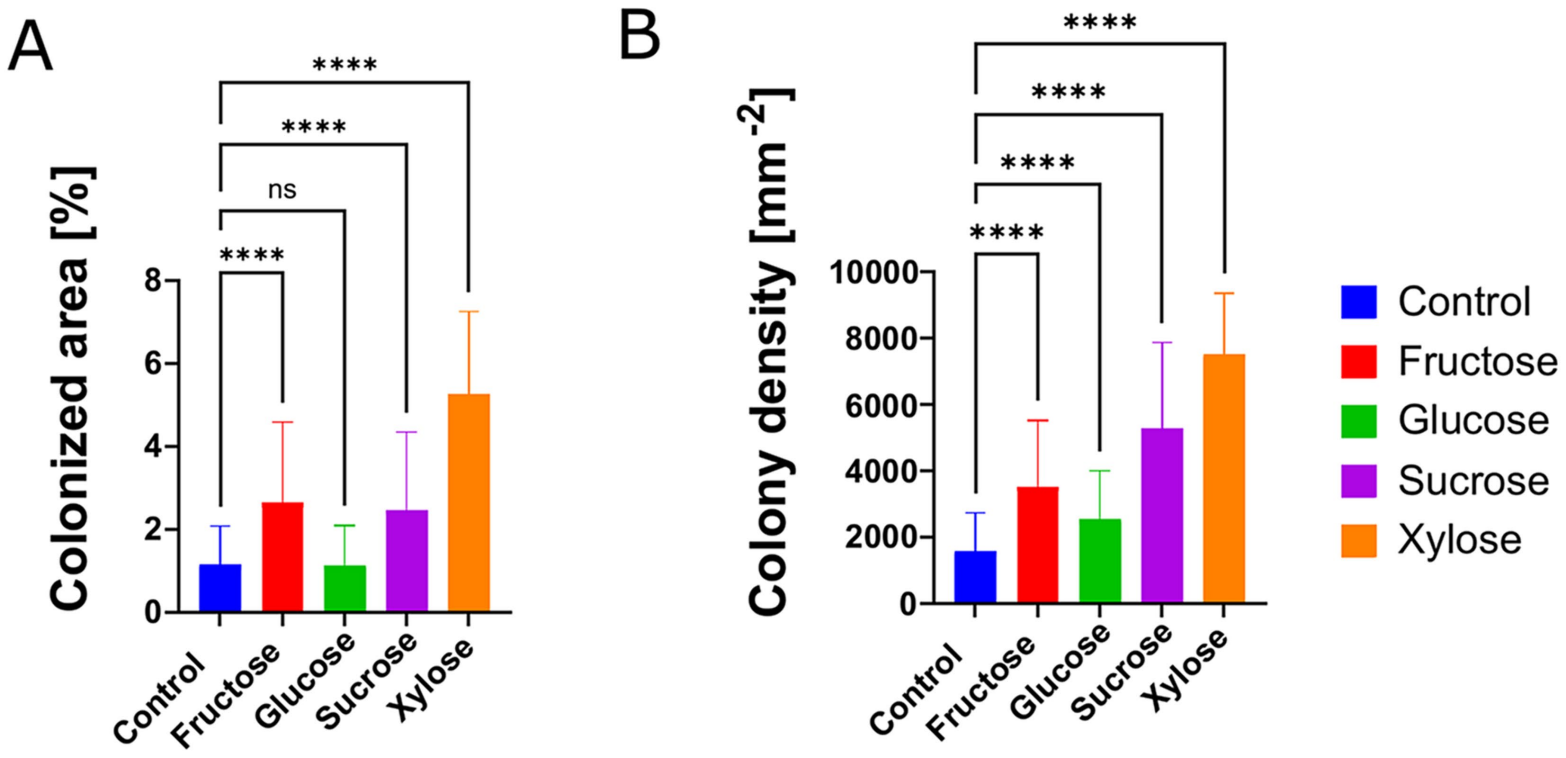
Sugar supplementation enhances biofilm formation in *S. acidophilus*ᵀ. Cultures were grown under static conditions on sulfur prills and supplemented with fructose, sucrose, or xylose at a final concentration of 1 mM. Each condition was tested in triplicate with 256 images acquired and analyzed per replicate. Samples were collected after 96 h. Biofilm formation was quantified using a high-throughput image analysis workflow: **(A)** colony density and **(B)** percentage of colonized area. Statistical significance was determined using a Kruskal–Wallis test followed by Dunn’s *post hoc* test. (****) = *p* < 0.0001.

To evaluate the effect of sugar supplementation on biofilm development, static cultures were supplemented with individual sugars at a final concentration of 1 mM and compared to an unsupplemented control (Figure 2). Static incubation was chosen to favor surface attachment and biofilm formation. Supplementation with xylose, fructose and sucrose resulted in a significant increase in both colony density and the percentage of colonized surface area. In contrast, glucose supplementation led to a significant increase in colony density but did not result in a corresponding increase in the colonized area. These results indicate that fructose, xylose and sucrose significantly enhance *S. acidophilus*^T^ biofilm formation on S^0^ (Figure 2).

Since nucleobase transporters are encoded in *S. acidophilus*^T^, we also teste addition of DNA. Static cultures of *S. acidophilus*^T^ supplemented with extracellular DNA at a final concentration of 1 ng/μL exhibited a significant increase in biofilm formation (Supplementary Figures 1, 2).

### 3.3 Fluorescence lectin binding analysis indicates increased amounts of extracellular polysaccharides

An initial lectin screening using FITC- or TRITC-conjugated lectins was performed to evaluate the binding capacity of lectins to *S. acidophilus*ᵀ biofilms grown under basal culture conditions (without sugar supplementation). The following lectins showed positive binding: ConA, ECA, GNA, HHA, MPA, and WGA (Table 2). The strongest signals were obtained with ConA and HHA, indicating a high content of mannose-containing residues within the biofilm matrix (Table 2).

**Table 2:** Lectin screening for *S. acidophilus*^T^.

Lectin	Specificity	Affinity
AAL	α-Fucose	−
ConA	α-D-mannose, α-D glucose	+
ECA	Galactose, (β 1,4) N-Acetylglucosamine	+
GNA	Mannose	+
GS-I	Melibiose, α-D galactose	−
HHA	α-(1,3), α-(1,2) mannose	+
MPA	N-acetylgalactosamine, Galactose	+
WGA	N-acetylglucosamine	+

From left to right are shown: the lectins used, their specificity, and finally the binding capacity of each lectin. Lectin specificities were obtained from Neu and Kuhlicke (2017).

**Figure 3:**
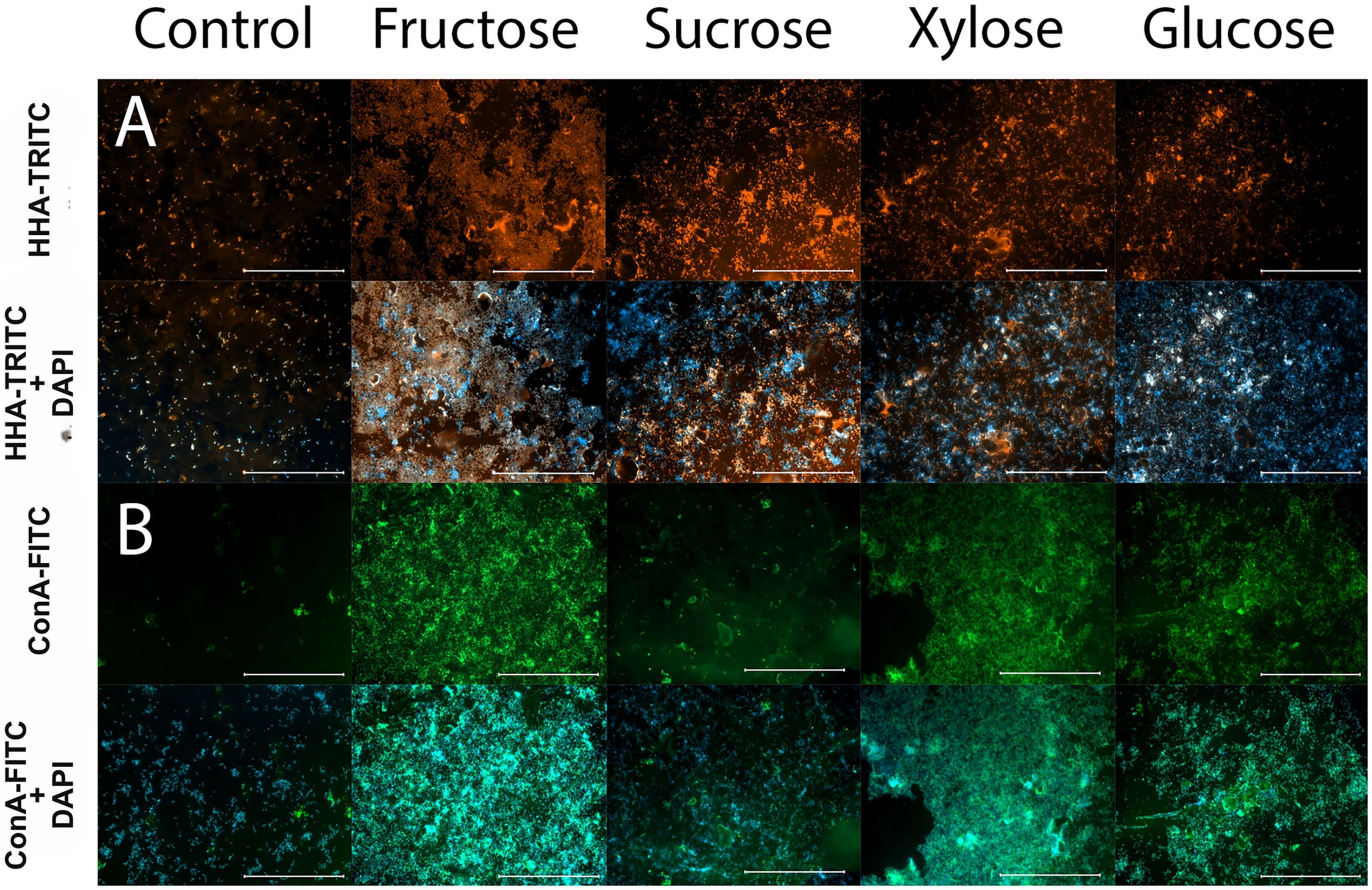
Sugar supplementation enhances extracellular polysaccharide abundance in the *Sulfobacillus acidophilus*ᵀ biofilm matrix as revealed by FLBA. **(A)** *S. acidophilus*ᵀ biofilms stained with the HHA-TRITC lectin, which binds to internal and terminal mannose residues. The upper panel shows the TRITC channel, and the lower panel show merged DAPI-TRITC images. **(B)** *S. acidophilus*ᵀ biofilms stained with ConA-FITC, which binds glucose and mannose residues. The upper panels show the FITC channel, and the lower panel shows the merged DAPI-FITC images.

As stated, *Canavalia ensiformis* hemagglutinin (Concanavalin A, ConA), which binds mannose and glucose residues, and *Hippeastrum* hybrid agglutinin (HHA), which recognizes (terminal mannose and mannose core trisaccharides) produced the strongest and most consistent binding signals under chemolithotrophic cultivation conditions. These lectins were therefore selected for further analyses of *S. acidophilus*ᵀ biofilm grown with sugar supplementation. Supplementation with fructose, glucose, xylose and sucrose, was tested. FLBA pattern of biofilm matrix remained similar across all conditions, indicating that the addition of different sugars did not markedly alter polysaccharide structure. However, a visible increase in extracellular polysaccharide abundance within the biofilm matrix was observed upon sugar supplementation, consistent with the quantitative increase in biofilm formation (Figure 3; Table 3). Overall, FLBA results indicate that the extracellular PS matrix of. *S. acidophilus*^T^ is mainly composed of mannose and glucose residues, with minor contributions from galactose and N-acetylated sugars. In combination with the quantified increase in biofilm formation, these results suggest that sugar supplementation production and accumulation of extracellular PS within the matrix, and their overall composition remains largely unchanged.

**Table 3:** Lectin binding intensity increased when *S. acidophilus^T^* biofilms were grown with sugar-supplementation.

Lectin	Specificity	Control	Fructose	Sucrose	Xylose	Glucose
HHA	α-(1,3), α-(1,2) mannose	+	+++	+++	+++	++
ConA	α-D-mannose, α-D glucose	+	+++	++	+++	+++

Lectin binding was evaluated semi-quantitatively using a three-level scale: ‘+’ indicates weak but detectable binding ‘++’ indicates moderate binding, and ‘+++’ represents strong binding. The scoring was based on relative fluorescence intensity observed in EFM images.

### 3.4 Sugar supplementation increases planktonic growth when cultures are grown under agitation

**Figure 4:**
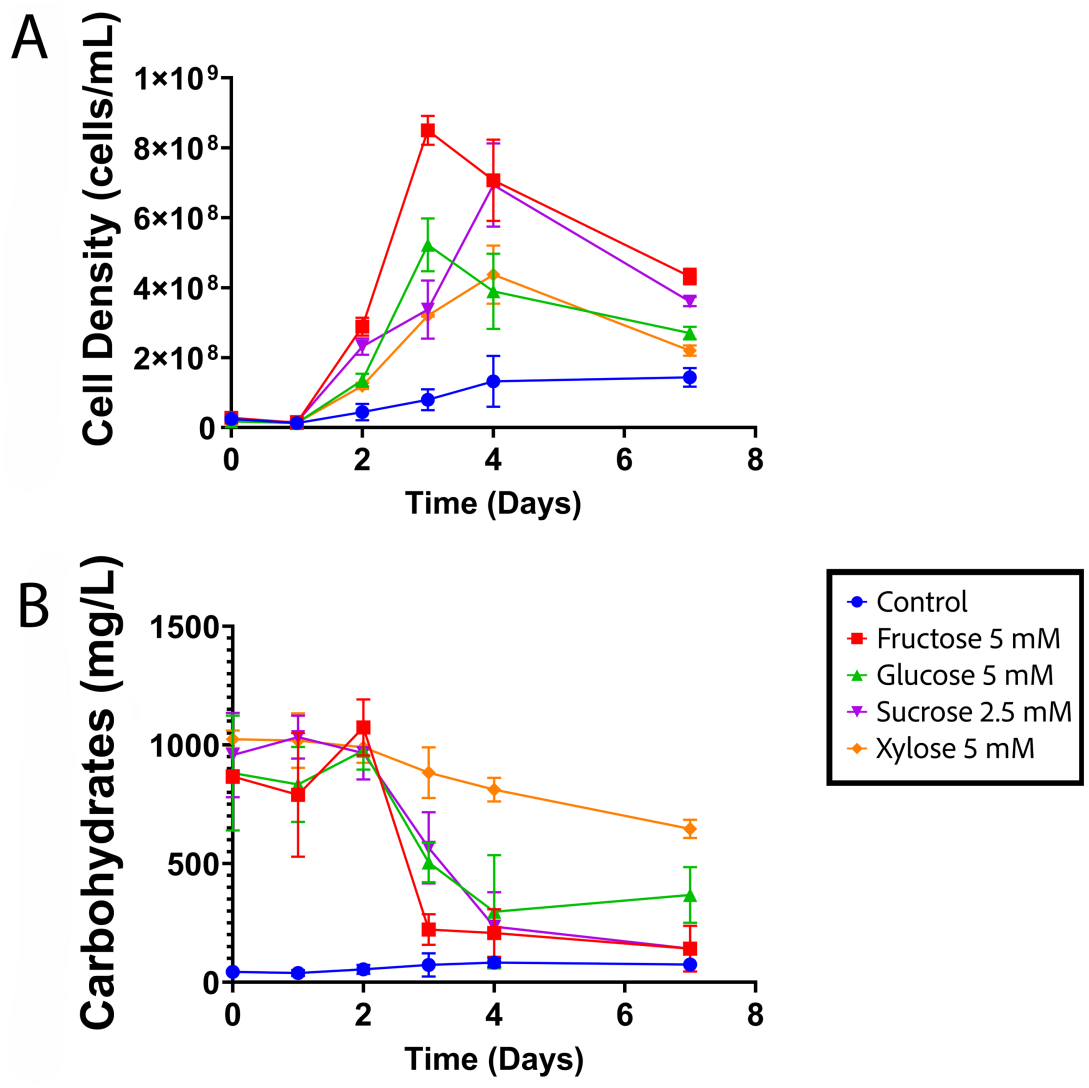
Sugar supplementation with fructose, glucose, xylose, or sucrose significantly enhances planktonic growth of *S. acidophilus*ᵀ in shaken cultures. Cultures were supplemented with glucose, fructose, or xylose at a final concentration of 5 mM, or sucrose at 2.5 mM, and grown in shaken flasks with elemental sulfur powder as the energy source. All conditions were tested in biological triplicates. Cell density **(A)** data were analyzed using a two-way ANOVA in GraphPad Prism. No significant differences were observed during the first 24 h of incubation. However, after 48 h, all sugar-supplemented cultures exhibited significantly higher cell densities than the non-supplemented control. Total carbohydrate concentrations in culture supernatants **(B)** progressively decreased over time, reflecting active sugar uptake and utilization by the cells.

Supplementation with fructose, glucose, xylose, or sucrose significantly increased the planktonic cell densities in *S. acidophilus*ᵀ cultures grown in agitated flasks compared with sugar-free controls (Figure 4A) The increase in biomass was accompanied by a progressive decrease in sugar concentrations in the culture medium, indicating active sugar uptake and utilization. Fructose and sucrose were almost completely depleted within 96 h of incubation (Figure 4B). In contrast, xylose was not fully consumed after 168 h, although a clear increase in cell density was still observed. Notably, this growth-promoting effect was not detected in cultures incubated under static conditions (not shown).

### 3.5 HPLC-ESI-MS/MS reveals enhanced polysaccharide production with conserved composition in *S. acidophilus*^T^

**Figure 5:**
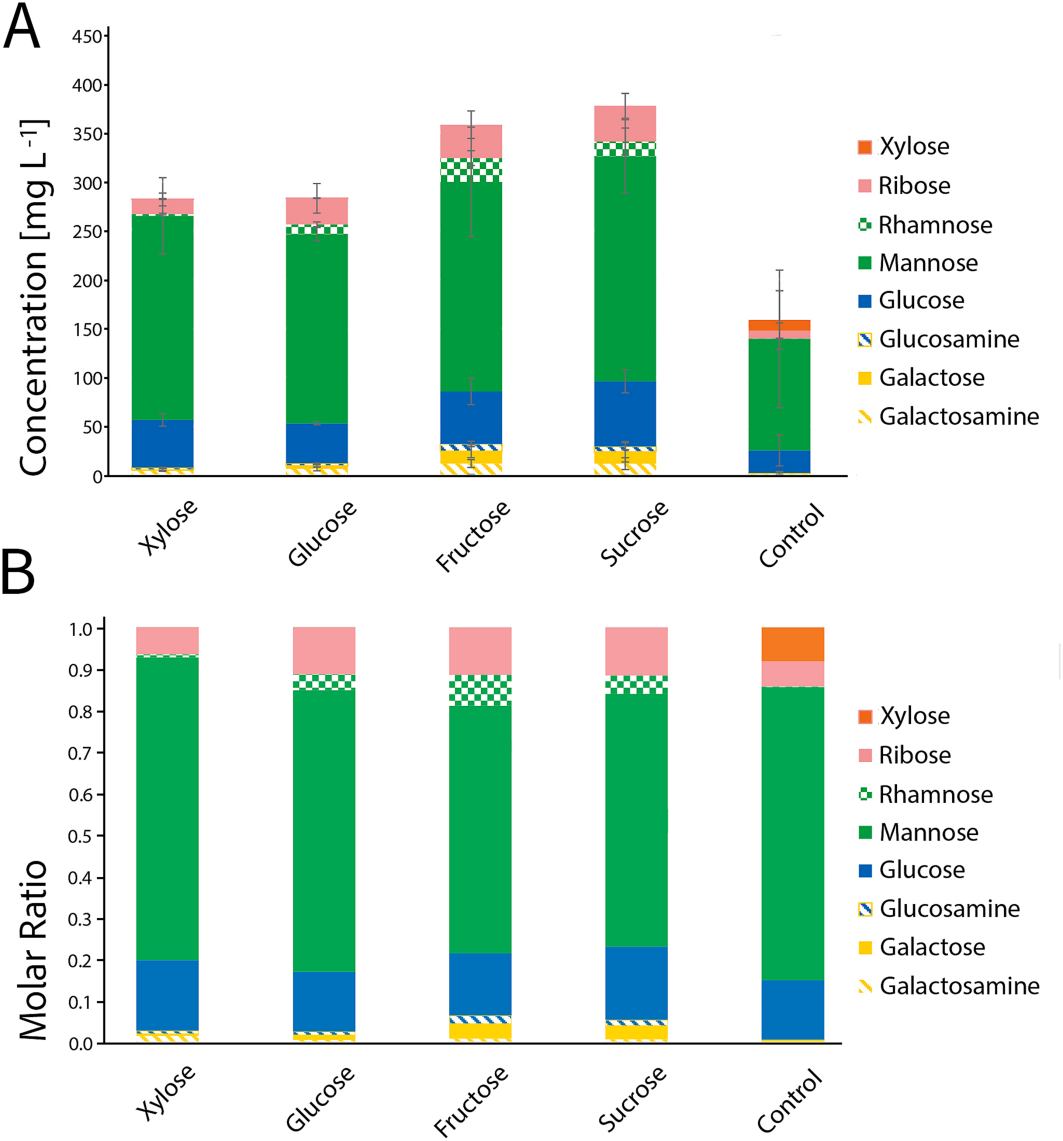
Sugar supplementation increases soluble polysaccharide production by *S. acidophilus*^T^, without altering polysaccharide composition. **(A)** Normalized concentration and relative abundance of soluble PS produced by *S. acidophilus*^T^ cultivated in shaken flasks under basal conditions or supplemented with xylose, glucose, fructose, or sucrose. Bars represent the total PS concentration, while stacked segments indicate the contribution of individual monosaccharides. Error bars correspond to the standard deviation of three biological replicates. Sugar supplementation resulted in a pronounced increase in total PS production. **(B)** Relative monosaccharide composition of soluble PS produced by *S. acidophilus*^T^ under the same conditions. The relative molar proportion of individual monosaccharides remained highly consistent across every tested condition. All components were analyzed and quantified via HPLC-ESI-MS/MS.

To quantify the effects of sugar supplementation on soluble PS production by *S. acidophilus*^T^, PS were recovered from the culture supernatants under each condition and analyzed by HPLC-ESI-MS/MS. Although minor variations in PS yields were observed among the different sugar-supplemented cultures, the addition of fructose, glucose, xylose or sucrose resulted in a pronounced increase in total PS production compared with the non-supplemented control (Figure 5A).

The monosaccharide composition of PS produced by *S. acidophilus*^T^ remained largely consistent across all supplementation conditions. The PS backbone was primarily composed of mannose, with glucose present at lower relative abundance. In addition, residues of ribose, galactose, glucosamine and galactosamine were detected under all tested conditions. Rhamnose residues were detected only in sugar-supplemented cultures. Notably, xylose residues were detected in the non-supplemented controls but were absent in cultures supplemented with xylose. Residues of ribose, galactose, glucosamine and galactosamine were detected under all tested conditions (Figure 5B).

## 4 Discussion

Our bioinformatic analysis revealed that *Sulfobacillus* species encode a diverse repertoire of sugar transporters, which may play a central role in their nutritional versatility and contribute to an increased intracellular supply of sugar monomers for extracellular PS synthesis during biofilm formation. In addition to sugar transporters, we also identified multiple transport systems predicted to mediate the uptake of nucleosides and nucleobases. The potential contribution of these transporters to cellular growth and biofilm development is discussed below. Notably, the number of putative mono- and disaccharide transporters identified in *S. acidophilus*^T^ and *S. thermosulfidooxidans*^T^ is substantially higher than that reported for other acidophilic genera (Alegría and Vera, unpublished data). This finding is consistent with the metabolic flexibility that distinguishes *Sulfobacillus* from chemolithoautotrophic bioleaching bacteria. Their capability of mixotrophic growth favors carbon cycling in bioleaching microbial systems, since the use of organic substrates may be particularly advantageous in nutrient-poor bioleaching consortia, where organic compounds produced by neighboring species or dead biomass can be recycled. For example, species of *Acidithiobacillus* and *Leptospirillum* secrete glycolic acid, which is toxic to several bioleaching microorganisms, whereas *Sulfobacillus* species can metabolize this compound (Ñancucheo and Johnson, 2010).

As described above, a complete ABC CUT-1 transporter typically consists of two TM subunits, one SBP, and one NBP. In this study, no genes encoding NBP domains were detected in the vicinity of the five putative ABC CUT-1 transporters identified in the genomes of *S. acidophilus*^T^ and *S. thermosulfidooxidans*^T^. Instead, a single NBP CUT-1 subunit was found to be encoded at a distant genomic locus, suggesting that this subunit might be shared among multiple ABC CUT1 transport systems. This genomic organization, in which the NBP subunit is encoded separately from the remaining components of the ABC CUT-1 transporter, represents a common feature in several bacterial species (Leisico et al., 2020). The putative NBP sequences identified in both *Sulfobacillus* species were orthologous to each other and are homologous to the well-characterized MalK1 ATP-binding protein from *Thermus thermophilus* and *Streptococcus mutans.* Notably, in *S. mutans*, MalK1 has been shown to be interchangeable among several sugar-uptake ABC systems (Webb et al., 2008), supporting the concept of functional sharing. Similarly, in *T. thermophilus*, MalK1 has been experimentally demonstrated to energize two distinct ABC transporters and is encoded distantly from the operons containing the remaining transporter components (Chevance et al., 2006).

Interestingly, xylose supplementation significantly enhanced biofilm formation and EPS production in *S. acidophilus*^T^. This observation is consistent with the *in-silico* identification of a putative xylose-specific ABC transporter in this species and suggests that this system may mediate xylose uptake. Support for the functionality of this transporter comes from previous studies in *Sulfolobus acidocaldarius* MW001, where transcriptomic analyses showed that D-xylose supplementation led to increased expression of a xylose ABC transporter relative to basal conditions (Wagner et al., 2018). Notably, the SBP sequence of the xylose ABC transporter described in *S. acidocaldarius* is identical to the SBP identified in the genome of *S. acidophilus*ᵀ in this study. In bacteria, D-xylose is converted to D-xylulose by xylose isomerase, then D-xylulose is subsequently phosphorylated by xylulose kinase (EC 2.7.1.17). The resulting D-xylulose-5-phosphate can enter the pentose phosphate pathway to generate ribulose-5-phosphate or be further metabolized into intermediates of glycolysis (Jeffries, 1983). Consistent with this metabolic framework, genes encoding homologs of xylose isomerase and xylulose kinase were identified in the genomic vicinity of the putative xylose ABC transporter in *S. acidophilus*ᵀ, suggesting that these enzymes likely function in downstream xylose metabolism following uptake.

Our *in-silico* analyses also identified a putative ABC CUT-2 type nucleoside transporter in both, *S. acidophilus*^T^ and *S. thermosulfidooxidans*^T^ genomes, along with multiple NCS-1 transporters predicted to mediate nucleobases uptake. The import of nucleosides and nitrogenous bases is a widespread trait among bacteria. For example, *Escherichia coli* BL21 (DE3) can grow using nucleosides as the sole source of carbon and nitrogen (Xie et al., 2004), and similar uptake and utilization strategies have been described in *Bacillus subtilis* (Johansen et al., 2003) and *Lactococcus lactis* (Martinussen et al., 2010). In these organisms, nucleosides are typically imported via ABC-type transporters, whereas nucleobases are taken up through NCS- or MFS-type systems (Martinussen et al., 2010; Weyand et al., 2008). Nucleotides and nucleobases in the extracellular environment can originate from nucleic acid degradation, a process that is particularly prevalent in acidic environments (pH < 3), where sulfobacilli thrives. Nucleobases can be directly imported and incorporated into *de novo* nucleotide synthesis pathways, whereas extracellular nucleotides are first dephosphorylated by secreted nucleosidases, allowing their uptake as nucleosides. Once inside the cell, nucleosides can either be rephosphorylated into nucleotides by specific kinases or cleaved into their constituent nucleobase and sugar moieties by nucleoside phosphorylases (Kilstrup et al., 2005).

The putative ABC transporters identified in *Sulfobacillus* are homologous to the NupNOPQ nucleoside transport system described in *B. subtilis* (Belitsky and Sonenshein, 2011). In the genomic vicinity of these transport systems, genes encoding putative ribose or nucleoside kinases (SMC05777.1 and AEW06307.1) as well as putative nucleotide glucosidases (SMC05774.1 and AEW06308.1) were identified. These kinases could phosphorylate the nucleosides imported via the NupNOPQ-like transporter, thereby channeling them directly into nucleic acid biosynthesis and reducing the energetic cost associated with de novo nucleotide synthesis. In parallel, the putative nucleotide glucosidases may cleave imported nucleosides into their nitrogenous base and deoxyribose components, providing additional metabolic flexibility for *Sulfobacillus*.

DNA supplementation also enhanced biofilm formation by *S. acidophilus*^T^ and *S. thermosulfidooxidans*^T^ under static growth conditions (Supplementary Figure 1). DNA structure is highly sensitive to pH: at values below three, the double helix denatures into single strands. Under extremely acidic conditions, nucleobases undergo hydrolysis, leading to cleavage of the N-glycosidic bond between the base and the sugar moiety. Progressive base loss ultimately results in strand breaks (Shapiro, 1981). Consequently, exogenous DNA added to the cultures was likely degraded by the acidic medium, indirectly supplying the cells with a mixture of nucleosides and free nitrogenous bases. Further analyses are required to determine whether DNA supplementation also affects planktonic growth and extracellular PS production.

FLBA and HPLC analyses indicate that the extracellular PS produced by *Sulfobacillus* possesses a mannose-dominant backbone, with a minor contribution from glucose. While the overall monosaccharide composition remained consistent across all tested conditions, sugar supplementation markedly increased total PS production. This enhancement was evident both in the greater incorporation of sugar residues into the EPS matrix, as observed by FLBA, and in the elevated concentration of soluble PS in culture supernatants, as determined by chromatographic analysis.

Furthermore, sugar addition also led to an increase in planktonic cell counts in agitated flask cultures; however, this effect was not observed under static conditions during biofilm formation assays. Therefore, the observed enhancement in biofilm formation under static conditions is likely attributable to a higher proportion of cells attaching to surfaces rather than to an increase in total cell abundance. The absence of planktonic growth response under static conditions may be explained by limited aeration and the reduced availability of sulfur surface area, as prills provide fewer contact points compared to sulfur powder.

Total carbohydrate measurements using the Dubois method confirmed that *Sulfobacillus* cells actively consumed the supplemented sugars. Among the sugars tested, fructose was depleted most rapidly, which may reflect preferential uptake via the putative PTS transporter, consistent with the known substrate preference of PTS systems in other bacteria. In contrast, xylose was the least consumed sugar. Nevertheless, xylose supplementation still enhanced PS production, biofilm formation and planktonic growth, suggesting that xylose may be more efficiently incorporated into extracellular PS than fructose or glucose. These observations point to a potentially relevant role for pentoses in *Sulfobacillus* metabolism, which warrants further investigation.

In summary, our combined *in silico* and experimental analysis indicates that *Sulfobacillus* can import environmental sugars and channel them into extracellular PS biosynthesis. Sugar supplementation increased biofilm formation by *S. acidophilus*^T^ on sulfur pearls, and this effect correlated with enhanced PS production as revealed by FLBA and HPLC analyses. These findings suggest that increased cell adhesion is supported by augmented extracellular PS synthesis.

Overall, these results highlight the metabolic versatility of *Sulfobacillus* spp. and provide a foundation for studying biofilm formation by bioleaching bacteria *in situ*. This metabolic complexity is reflected in the diversity of transporters and the subsequent processing of imported sugars through multiple, as-yet-uncharacterized pathways. As a ferrous iron/sulfur oxidizer capable of growth in sugar-free media, *Sulfobacillus* represents an ideal model to study sugar uptake and its integration into PS biosynthesis with high carbon-use efficiency. Building on these findings, future work will focus on further characterizing these pathways in *S. acidophilus* and developing an integrated model linking carbohydrate transport, catabolism, and their contribution to EPS production.

## Data Availability

The original contributions presented in the study are included in the article/Supplementary material, further inquiries can be directed to the corresponding authors.

## Footnotes

1
https://tcdb.org/

2
https://dtu.biolib.com/DeepTMHMM

3
https://dtu.biolib.com/SignalP-6

4
https://www.ncbi.nlm.nih.gov/cdd/

5
http://pfam.xfam.org/

6
https://prosite.expasy.org/scanprosite/

7
https://meme-suite.org/meme/doc/fimo.html

8
https://rast.nmpdr.org/

## References

[ref1] Aziz R. K. Bartels D. Best A. A. DeJongh M. Disz T. Edwards R. A. . (2008). The RAST server: rapid annotations using subsystems technology. BMC Genomics 9:75. doi: 10.1186/1471-2164-9-75, 18261238 PMC2265698

[ref2] Belitsky B. R. Sonenshein A. L. (2011). CodY-mediated regulation of guanosine uptake in *Bacillus subtilis*. J. Bacteriol. 193, 6276–6287. doi: 10.1128/jb.05899-11, 21926227 PMC3209203

[ref3] Bellenberg S. Buetti-Dinh A. Galli V. Ilie O. Herold M. Christel S. . (2018). Automated microscopic analysis of metal sulfide colonization by acidophilic microorganisms. Appl. Environ. Microbiol. 84, e01835–e01818. doi: 10.1128/AEM.01835-18, 30076195 PMC6182891

[ref4] Bellenberg S. Leon-Morales C. F. Sand W. Vera M. (2012). Visualization of capsular polysaccharide induction in *Acidithiobacillus ferrooxidans*. Hydrometallurgy 129, 82–89. doi: 10.1016/j.hydromet.2012.09.002

[ref5] Caldwell P. E. MacLean M. R. Norris P. R. (2007). Ribulose bisphosphate carboxylase activity and a Calvin cycle gene cluster in *Sulfobacillus* species. Microbiology 153, 2231–2240. doi: 10.1099/mic.0.2007/006262-0, 17600067

[ref6] Chevance F. F. Erhardt M. Lengsfeld C. Lee S. J. Boos W. (2006). Mlc of *Thermus thermophilus*: a glucose-specific regulator for a glucose/mannose ABC transporter in the absence of the phosphotransferase system. J. Bacteriol. 188, 6561–6571. doi: 10.1128/jb.00715-06, 16952948 PMC1595481

[ref7] Christel S. Herold M. Bellenberg S. Buetti-Dinh A. El Hajjami M. Pivkin I. V. . (2018). Weak iron oxidation by *Sulfobacillus thermosulfidooxidans* maintains a favorable redox potential for chalcopyrite bioleaching. Front. Microbiol. 9:3059. doi: 10.3389/fmicb.2018.03059, 30631311 PMC6315122

[ref8] De Castro E. Sigrist C. J. Gattiker A. Bulliard V. Langendijk-Genevaux P. S. Gasteiger E. . (2006). ScanProsite: detection of PROSITE signature matches and ProRule-associated functional and structural residues in proteins. Nucleic Acids Res. 34, W362–W365. doi: 10.1093/nar/gkl124, 16845026 PMC1538847

[ref9] Drew D. North R. A. Nagarathinam K. Tanabe M. (2021). Structures and general transport mechanisms by the major facilitator superfamily (MFS). Chem. Rev. 121, 5289–5335. doi: 10.1021/acs.chemrev.0c00983, 33886296 PMC8154325

[ref10] Dubois M. Gilles K. A. Hamilton J. K. Rebers P. A. Smith F. (1951). A colorimetric method for the determination of sugars. Nature 168:167. doi: 10.1038/168167a0, 14875032

[ref11] Flemming H. C. Wingender J. Szewzyk U. Steinberg P. Rice S. A. Kjelleberg S. (2016). Biofilms: an emergent form of bacterial life. Nat. Rev. Microbiol. 14, 563–575. doi: 10.1038/nrmicro.2016.94, 27510863

[ref12] Grant C. Bailey T. L. Noble W. S. (2011). FIMO: scanning for occurrences of a given motif. Bioinformatics 27, 1017–1018. doi: 10.1093/bioinformatics/btr064, 21330290 PMC3065696

[ref13] Gutknecht R. Beutler R. Garcia-Alles L. F. Baumann U. Erni B. (2001). The dihydroxyacetone kinase of *Escherichia coli* utilizes a phosphoprotein instead of ATP as phosphoryl donor. EMBO J. 20, 2480–2486. doi: 10.1093/emboj/20.10.2480, 11350937 PMC125457

[ref14] Hallgren J. Tsirigos K. D. Pedersen M. D. Almagro Armenteros J. J. Marcatili P. Nielsen H. . (2022). DeepTMHMM predicts alpha and beta transmembrane proteins using deep neural networks. BioRxiv, 2022–2004. doi: 10.1101/2022.04.08.487609

[ref15] Jeckelmann J. M. Erni B. (2020). Transporters of glucose and other carbohydrates in bacteria. Pflugers Arch. - Eur. J. Physiol. 472, 1129–1153. doi: 10.1007/s00424-020-02379-0, 32372286

[ref16] Jeffries T. W. (1983). Utilization of xylose by bacteria, yeasts, and fungi. Pentoses and lignin. Adv. Biochem. Eng. Biotechnol. 27, 1–32. doi: 10.1007/BFb0009101 6437152

[ref17] Johansen L. E. Nygaard P. Lassen C. Agersø Y. Saxild H. H. (2003). Definition of a second *Bacillus subtilis* pur regulon comprising the pur and xpt-pbuX operons plus pbuG, nupG (yxjA), and pbuE (ydhL). J. Bacteriol. 185, 5200–5209. doi: 10.1128/jb.185.17.5200-5209.2003, 12923093 PMC181001

[ref19] Justice N. B. Norman A. Brown C. T. Singh A. Thomas B. C. Banfield J. F. (2014). Comparison of environmental and isolate *Sulfobacillus* genomes reveals diverse carbon, sulfur, nitrogen, and hydrogen metabolisms. BMC Genomics 15:1107. doi: 10.1186/1471-2164-15-1107, 25511286 PMC4378227

[ref20] Kilstrup M. Hammer K. Ruhdal Jensen P. Martinussen J. (2005). Nucleotide metabolism and its control in lactic acid bacteria. FEMS Microbiol. Rev. 29, 555–590. doi: 10.1016/j.femsre.2005.04.006 15935511

[ref22] Leisico F. Godinho L. M. Gonçalves I. C. Silva S. P. Carneiro B. Romão M. J. . (2020). Multitask ATPases (NBDs) of bacterial ABC importers type I and their interspecies exchangeability. Sci. Rep. 10:19564. doi: 10.1038/s41598-020-76444-0, 33177617 PMC7658222

[ref23] Marchler-Bauer A. Bryant S. H. (2004). CD-search: protein domain annotations on the fly. Nucleic Acids Res. 32, W327–W331. doi: 10.1093/nar/gkh454, 15215404 PMC441592

[ref24] Martinussen J. Sørensen C. Jendresen C. B. Kilstrup M. (2010). Two nucleoside transporters in *Lactococcus lactis* with different substrate specificities. Microbiology 156, 3148–3157. doi: 10.1099/mic.0.039818-0, 20595258

[ref25] Merino M. P. Andrews B. A. Parada P. Asenjo J. A. (2016). Characterization of *Ferroplasma acidiphilum* growing in pure and mixed culture with *Leptospirillum ferriphilum*. Biotechnol. Prog. 32, 1390–1396. doi: 10.1002/btpr.2340, 27535541

[ref26] Mistry J. Chuguransky S. Williams L. Qureshi M. Salazar G. A. Sonnhammer E. L. . (2021). Pfam: the protein families database in 2021. Nucleic Acids Res. 49, D412–D419. doi: 10.1093/nar/gkaa913, 33125078 PMC7779014

[ref27] Ñancucheo I. Johnson D. B. (2010). Production of glycolic acid by chemolithotrophic iron-and sulfur-oxidizing bacteria and its role in delineating and sustaining acidophilic sulfide mineral-oxidizing consortia. Appl. Environ. Microbiol. 76, 461–467. doi: 10.1128/AEM.01832-09, 19933342 PMC2805229

[ref28] Neu T. R. Kuhlicke U. (2017). Fluorescence lectin bar-coding of glycoconjugates in the extracellular matrix of biofilm and bioaggregate forming microorganisms. Microorganisms 5:5. doi: 10.3390/microorganisms5010005, 28208623 PMC5374382

[ref31] Norris P. R. Clark D. A. Owen J. P. Waterhouse S. (1996). Characteristics of *Sulfobacillus acidophilus* sp. nov. and other moderately thermophilic mineral-sulphide-oxidizing bacteria. Microbiology 142, 775–783. doi: 10.1099/00221287-142-4-775, 8936305

[ref32] Panyushkina A. Muravyov M. (2023). New features of acidophilic bacteria of the genus *Sulfobacillus*: polysaccharide biosynthesis and degradation pathways. Minerals 13:255. doi: 10.3390/min13020255

[ref33] Quistgaard E. M. Löw C. Guettou F. Nordlund P. (2016). Understanding transport by the major facilitator superfamily (MFS): structures pave the way. Nat. Rev. Mol. Cell Biol. 17, 123–132. doi: 10.1038/nrm.2015.25, 26758938

[ref34] Rossoni S. Beard S. Segura-Bidermann M. I. Duarte-Ramírez J. Osorio F. K. Varas-Godoy M. . (2024). Membrane vesicles in *Acidithiobacillia* class extreme acidophiles: influence on collective behaviors of '*Fervidacidithiobacillus caldus*'. Front. Microbiol. 14:1331363. doi: 10.3389/fmicb.2023.1331363, 38344243 PMC10853474

[ref35] Rühmann B. Schmid J. Sieber V. (2014). Fast carbohydrate analysis via liquid chromatography coupled with ultraviolet and electrospray ionization ion trap detection in 96-well format. J. Chromatogr. A 1350, 44–50. doi: 10.1016/j.chroma.2014.05.014, 24861788

[ref36] Saier M. H. Jr. (2015). The bacterial phosphotransferase system: new frontiers 50 years after its discovery. J. Mol. Microbiol. Biotechnol. 25, 73–78. doi: 10.1159/000381215, 26159069 PMC4512285

[ref37] Saier M. H. Reddy V. S. Moreno-Hagelsieb G. Hendargo K. J. Zhang Y. Iddamsetty V. . (2021). The transporter classification database (TCDB): 2021 update. Nucleic Acids Res. 49, D461–D467. doi: 10.1093/nar/gkaa1004, 33170213 PMC7778945

[ref38] Schneider E. (2001). Abc transporters catalyzing carbohydrate uptake. Res. Microbiol. 152, 303–310. doi: 10.1016/S0923-2508(01)01201-3, 11421277

[ref39] Schneider E. Hunke S. (1998). ATP-binding-cassette (ABC) transport systems: functional and structural aspects of the ATP-hydrolyzing subunits/domains. FEMS Microbiol. Rev. 22, 1–20. doi: 10.1111/j.1574-6976.1998.tb00358.x, 9640644

[ref40] Shapiro R. (1981). “Damage to DNA caused by hydrolysis,” in Chromosome Damage and Repair, (Springer, New York, NY: NATO Advanced Study Institutes), 40, 3–18.

[ref41] Teufel F. Almagro Armenteros J. J. Johansen A. R. Gíslason M. H. Pihl S. I. Tsirigos K. D. . (2022). SignalP 6.0 predicts all five types of signal peptides using protein language models. Nat. Biotechnol. 40, 1023–1025. doi: 10.1038/s41587-021-01156-3, 34980915 PMC9287161

[ref42] Tsujibo H. Kosaka M. Ikenishi S. Sato T. Miyamoto K. Inamori Y. (2004). Molecular characterization of a high-affinity xylobiose transporter of *Streptomyces thermoviolaceus* OPC-520 and its transcriptional regulation. J. Bacteriol. 186, 1029–1037. doi: 10.1128/jb.186.4.1029-1037.2004, 14761997 PMC344215

[ref43] Vera M. Schippers A. Sand W. (2022). Progress in bioleaching: fundamentals and mechanisms of bacterial metal sulfide oxidation—part A. Appl. Microbiol. Biotechnol. 97, 7529–7541. doi: 10.1007/s00253-022-12168-7, 23720034

[ref44] Wagner M. Shen L. Albersmeier A. van der Kolk N. Kim S. Cha J. . (2018). *Sulfolobus acidocaldarius* transports pentoses via a carbohydrate uptake transporter 2 (CUT2)-type ABC transporter and metabolizes them through the aldolase-independent Weimberg pathway. Appl. Environ. Microbiol. 84, e01273–e01217. doi: 10.1128/AEM.01273-17, 29150511 PMC5772230

[ref45] Webb A. J. Homer K. A. Hosie A. H. (2008). Two closely related ABC transporters in *Streptococcus mutans* are involved in disaccharide and/or oligosaccharide uptake. J. Bacteriol. 190, 168–178. doi: 10.1128/jb.01509-07, 17965163 PMC2223742

[ref46] Weyand S. Shimamura T. Yajima S. Suzuki S. I. Mirza O. Krusong K. . (2008). Structure and molecular mechanism of a nucleobase–cation–symport-1 family transporter. Science 322, 709–713. doi: 10.1126/science.1164440, 18927357 PMC2885439

[ref47] Xie H. Patching S. G. Gallagher M. P. Litherland G. J. Brough A. R. Venter H. . (2004). Purification and properties of the *Escherichia coli* nucleoside transporter NupG, a paradigm for a major facilitator transporter sub-family. Mol. Membr. Biol. 21, 323–336. doi: 10.1080/09687860400003941, 15513740

[ref48] Zhang R. Hedrich S. Jin D. Breuker A. Schippers A. (2021). *Sulfobacillus harzensis* sp. nov., an acidophilic bacterium inhabiting mine tailings from a polymetallic mine. Int. J. Syst. Evol. Microbiol. 71:004871. doi: 10.1099/ijsem.0.004871, 34236956 PMC8489842

[ref49] Zhang R. Neu T. R. Zhang Y. Bellenberg S. Kuhlicke U. Li Q. . (2015). Visualization and analysis of EPS glycoconjugates of the thermoacidophilic archaeon *Sulfolobus metallicus*. Appl. Microbiol. Biotechnol. 99, 7343–7356. doi: 10.1007/s00253-015-6775-y, 26169631

