# Supplementary material for: Sugar supplementation enhances biofilm formation and extracellular polysaccharides production in Sulfobacillus acidophilus
Source: Front Microbiol. 2026 May 19;17:1838761. doi: 10.3389/fmicb.2026.1838761 (PMC13226494; doi:10.3389/fmicb.2026.1838761)
Supplement: Supplementary file 1 [file Supplementary_file_1.DOCX]

**
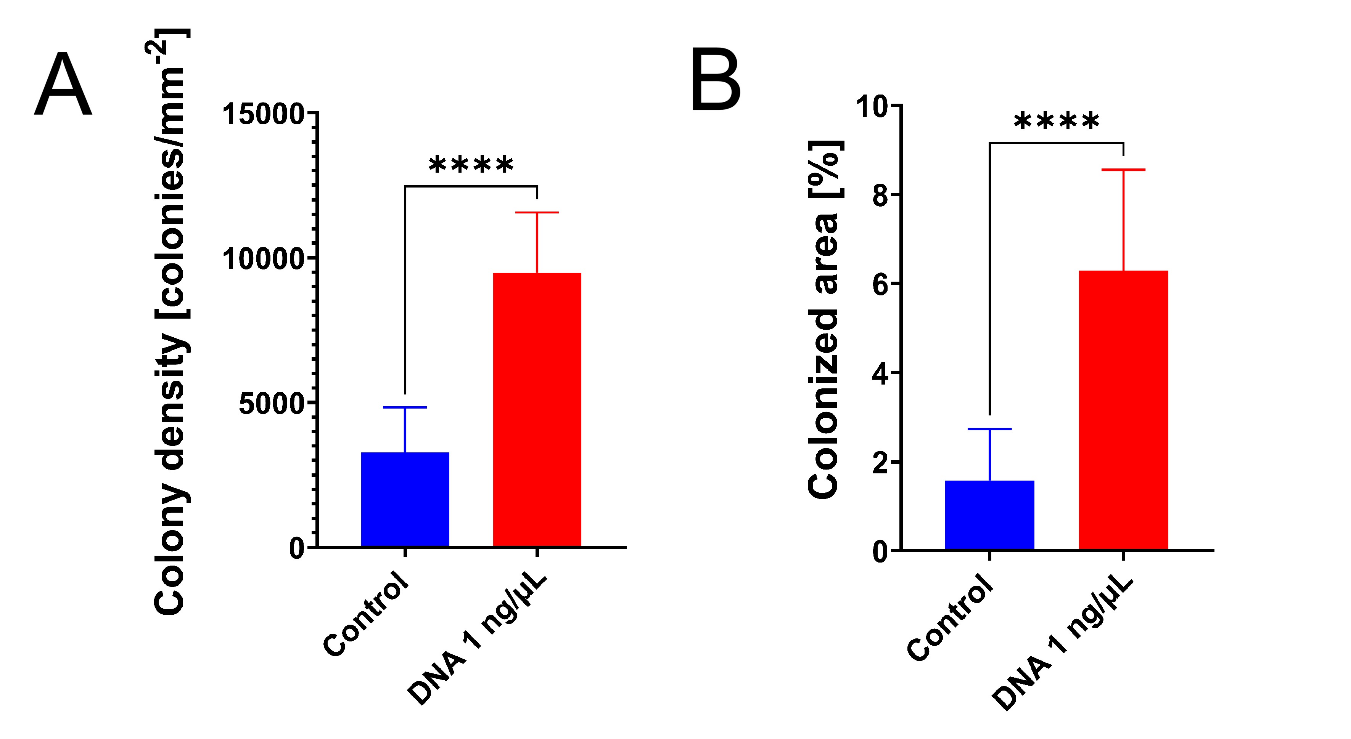
**

**Supplementary Figure 1. The addition of genomic DNA increases biofilm formation in *S.* *acidophilus*^T^**

Cultures were grown under static conditions on sulfur prills and DNA was added at a final concentration of 1 ng/µL. Every condition was tested in triplicate; 256 images were acquired and analyzed per replicate. Samples were collected after 96 h. Biofilm formation was quantified using a high-throughput image analysis workflow: (A) colony density and (B) percentage of colonized area. Statistical significance was determined using a Kruskal–Wallis test followed by Dunn’s post hoc test. (****) = p (****) = p < 0.0001.


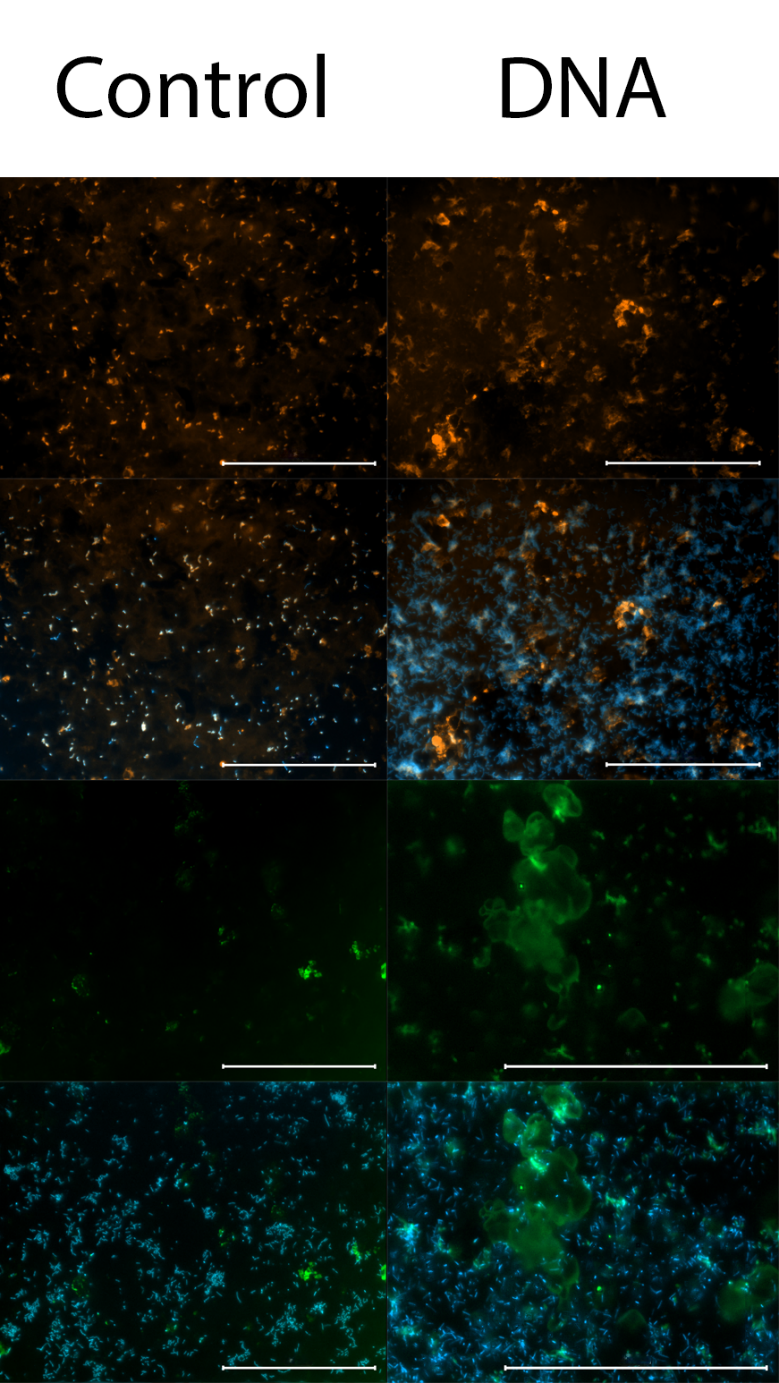


A

B

**Supplementary Figure 2. The addition of DNA enhances extracellular polysaccharides in *S. acidophilus*^T^ biofilms as evidenced by FLBA.**

(A) *S. acidophilus*^T^ biofilms labeled with HHA-TRITC lectin, which binds to mannose residues. The top panel displays the TRITC signal, while the bottom panel shows the merged DAPI–TRITC image. (B) *S. acidophilus*^T^ biofilms labeled with ConA-FITC lectin, which recognizes glucose and mannose residues. The top panel shows the FITC channel, and the bottom panel presents the merged DAPI–FITC signals. Size bars correspond to 20 µm.
